# Nurses' Readiness for Catastrophe Management and Its Relation to Their Organizational Commitment: Recommendations for Education

**DOI:** 10.1155/2024/5217371

**Published:** 2024-07-05

**Authors:** Aziza Z. Ali, Sameer A. Alkubati, Ahmad K. Al-Sadi, Wessam A. Elsayed, Shaimaa M. Nageeb, Nahed M. Saber, Sara F. Alenizi, Seham S. Alanazi, Mohannad J. Alkuwaisi, Laila A. Hamed

**Affiliations:** ^1^ Nursing Administration Department Faculty of Nursing University of Hail, Hail, Saudi Arabia; ^2^ Nursing Administration Department Faculty of Nursing Benha University, Benha, Egypt; ^3^ Medical Surgical Nursing Department College of Nursing University of Hail, Hail, Saudi Arabia; ^4^ Department of Nursing Faculty of Medicine and Health Sciences Hodeida University, Hodeida, Yemen; ^5^ Nursing Administration Department Faculty of Nursing Mansoura University, Mansoura, Egypt; ^6^ Psychiatric and Mental Health Nursing Faculty of Nursing Hail University, Hail, Saudi Arabia; ^7^ Psychiatric and Mental Health Nursing Faculty of Nursing Zagazig University, Zagazig, Egypt; ^8^ Maternal and Newborn Nursing Faculty of Nursing Beni-Suef University, Cairo, Egypt; ^9^ Maternal and Child Health Nursing Faculty of Nursing University of Hail, Hail, Saudi Arabia; ^10^ Medical Surgical Nursing Faculty of Nursing Zagazig University, Zagazig, Egypt

## Abstract

**Background:**

Catastrophes are challenging events for nations and health systems that require healthcare providers, especially nurses, to be prepared to respond effectively. Although nurses play a critical role in managing catastrophes and postcatastrophic situations, their preparedness is often inadequate and affected by their organizational commitment. Therefore, this study assessed nurses' preparedness for catastrophe management and its relationship with their organizational commitment.

**Methods:**

A cross-sectional correlational, descriptive design involving 286 conveniently sampled nurses was conducted in four public hospitals in Hail city. Data were collected using a questionnaire that compiled two tools: the Disaster Preparedness Evaluation Tool to assess nurses' preparedness for catastrophe management and the Organizational Commitment Scale to assess their attachment to their hospitals. Correlations between mean scores of nurses' knowledge, skills, and preparedness for postcatastrophe management and organizational commitment were tested using Spearman's correlation, with a significance level of <0.05.

**Results:**

Most nurses had low levels of knowledge (79.7%), skills (78.7%), and preparedness for postcatastrophe management (78.7% each). Meanwhile, 57.3% of nurses had low levels of affective commitment to their hospitals, compared to 78.7% for continuance and normative commitments. Statistically significant positive, moderate correlations were found between nurses' knowledge and skills in managing catastrophes (*r* = 0.512; *p* < 0.01) and knowledge and preparedness for postcatastrophe management (*r* = 0.492; *p* < 0.01), as well as nurses' skills and preparedness for postcatastrophe management (*r* = 0.533; *p* < 0.01). However, the nurses' level of organizational commitment was not significantly correlated with their knowledge, skills, or preparedness for postcatastrophe management.

**Conclusion:**

Nurses in Hail city are not adequately prepared to respond to and manage catastrophes and postcatastrophic situations, and they have low organizational commitments to their hospitals. Therefore, nursing education should integrate catastrophe management into the curricula, and hospital administrators should prioritize a supportive work environment that strengthens organizational commitment and provides ongoing education and regular training to improve nurses' preparedness for catastrophe management.

## 1. Introduction

Throughout history, catastrophic events have threatened entire nations and civilizations and resulted in lasting impacts on people's lives at various economic, social, and political levels. These impacts include death, disability, financial loss, and deterioration in living standards [1]. As catastrophes occur more frequently now than ever before, nurses must be well-prepared to respond and mitigate the negative effects they have on the affected population [2]. Despite efforts to prepare nurses for catastrophic events, they are still not adequately prepared to manage such situations. However, nurses' substantial contributions to global catastrophe relief efforts have been documented [3]. In 2015, natural catastrophes occurred in 99 countries, resulting in the displacement of millions of people, over 22,000 deaths, and an estimated economic loss of 70.3 billion USD [4].

A catastrophe is defined as any event that results in a dangerous and unstable situation that impacts a person, a group, or the entire society [5]. Unfavorable changes in human or environmental circumstances are called crises, especially when they occur unexpectedly and without warning. In a broader sense, a catastrophe is a difficult situation or an emergency. In other words, it is a “complex system” for the family, economy, and society that is disorganized [6].

Catastrophe nursing involves the systematic application of nursing knowledge and skills to address the challenges caused by catastrophes. It involves developing and implementing evidence-based practices to minimize the adverse health impacts of catastrophes and mitigate their life-threatening hazards [1]. A recent study showed that inadequate preparedness, inadequate formal training, limited empirical research, ethical and legal considerations, and concerns about personal safety and well-being during a catastrophe response are the most common challenges nurses face when responding to catastrophic events [7]. The lack of specificity in the extensive research in this area makes it necessary to identify the competencies required for the intended audiences and for different catastrophe situations and environments [8, 9].

Nurses' awareness of catastrophic events has increased in recent decades, but there remains a compelling need for further enhancement [10]. Many nurses lack the necessary psychological and educational preparedness to respond effectively to catastrophes [11–13]. Furthermore, much larger catastrophes are expected in the future, highlighting the need for greater awareness and preparedness. As a result, nurses should receive regular training in this area to keep their skills up-to-date [14, 15]. Since nurses play a vital role and are engaged in all phases of catastrophe response and management, it is important to provide them with the necessary knowledge, skills, and competencies and enhance their organizational commitment to effectively manage catastrophic events [16–18].

Organizational commitment refers to the psychological attachment that an employee feels toward a certain organization, which is characterized by alignment with its goals and values, as well as a strong desire to continue working there [19]. Nurses' organizational commitment is a critical factor in the success and effectiveness of healthcare facilities [20]. This commitment critically depends on the attitudes and behaviors of nurses, particularly when there are unmet expectations or limited resources [21]. Nurses who are highly committed to their organizations are more productive due to their dedication, loyalty, and sense of responsibility [6, 22].

Assessment of organizational commitment is important to understand staff nurses' commitment to the organization's mission and promote consistent work conduct [23], which can positively impact their performance, motivation, and attachment to the organization [24]. A recent study in Saudi Arabia by Al Harthi et al. [25] revealed certain strategies that could improve nursing management during disasters, such as providing personal protective equipment to nurses to reduce the risk of infection, developing assessment tools, and recognizing nurses for their efforts to reduce stress during a disaster [25]. More recently, Al Dulijand et al. [26] found lower rates of knowledge about incident command systems (49.9%) and mass casualty plans (56.1%) among healthcare workers in Saudi Arabia. The authors emphasized the need for targeted initiatives to improve their knowledge and training in these areas of disaster preparedness [26].

Several studies have focused on the social and environmental impacts of catastrophes [27, 28], but research on the relationship between nurses' organizational commitment and their readiness to manage catastrophes is limited. Although nurses are among the frontline responders during catastrophic events, their organizational commitment plays an important role in their ability to provide effective care to patients affected by these catastrophes [29]. In this regard, a positive relationship has been found between organizational commitment and nurses' ability to manage emergencies [22]. Therefore, this study aimed to assess nurses' preparedness for catastrophe management (knowledge, skills, and preparedness for postcatastrophe management) and its correlation with their level of organizational commitment.

### 1.1. Research Questions

1. What was the level of nurses' preparedness for catastrophe management?
2. What was the level of nurses' organizational commitment?
3. Was there a relationship between nurses' preparedness for catastrophe management and their organizational commitment?

## 2. Methods

### 2.1. Design

A cross-sectional correlational design was used. This study fulfilled the Strengthening the Reporting of Observational Studies in Epidemiology (STROBE) guideline [30].

### 2.2. Setting

This study was conducted in four main public hospitals in Hail city, Saudi Arabia: King Salman Hospital (500 beds), Hail General Hospital (136 beds), King Khaled Hospital (285 beds), and Maternity and Children's Hospital (200 beds).

### 2.3. Sampling Technique

A convenience sampling technique was used in this study. Considering a total of 1120 nurses in the four study hospitals, a minimum sample size of 286 was determined using the Raosoft_®_ sample size calculator (https://www.raosoft.com/samplesize.html), based on a 50% response distribution, a 95% confidence level, and a 5% accepted margin of error. The final sample consisted of 55 nurses from King Salman Hospital, 46 from Hail General Hospital, 85 from King Khaled Hospital, and 100 from Maternity and Children's Hospital.

The study included nurses who had a diploma or higher degree in nursing with at least one year of work experience, had worked as registered nurses in the selected hospitals, and agreed to voluntarily participate in the study. Assistant nurses, those with less than one year of work experience, and those who refused to give informed consent were excluded from the study.

### 2.4. Study Instruments

Data were collected using a questionnaire that compiled two tools: the Disaster Preparedness Evaluation Tool (DPET) and the Organizational Commitment Scale (OCS). Furthermore, the questionnaire included questions about the characteristics of nurses, including age, gender, nationality, qualification, work experience, number of hours worked per week, hospital affiliation, and involvement in real catastrophe management.

The Arabic version of the DPET, which was translated and validated by Al Khalaileh et al. [31], was used to assess nurses' perceptions of their preparedness for catastrophe management in three dimensions: knowledge, skills, and preparedness for postcatastrophe response and management [31]. It comprised the following 45 items: knowledge (13 items), skills (11 items), and postdisaster management (21 items). Nurses' responses to each item were rated on a 6-point Likert scale, ranging from 1 (strongly disagree) to 6 (strongly agree). Accordingly, the total scores for each participant ranged from 13 to 78 for knowledge, 11 to 66 for skills, and 21 to 126 for preparedness for postcatastrophe management, with higher scores indicating higher levels of preparedness. To calculate the mean total score for each dimension, the individual scores of participants for that dimension were summed and divided by the maximum possible score. The mean total scores were then converted into percentages by multiplying by 100. Based on the mean total percentage, the level of preparedness in each dimension was classified as low (<60%), moderate (60–75%), and high (>75%) [32–34].

The OCS was originally developed by Meyer and Allen in 1990 [35] and later updated in 1993 [36]. This scale comprised 18 items, divided evenly into three subscales: affective commitment, continuance commitment, and normative commitment. Nurses' responses to each item were rated on a 5-point Likert scale, ranging from 1 (strongly disagree) to 5 (strongly agree). To ensure consistent interpretation of responses, negatively worded items (3, 4, 5, and 13) were reverse scored. The mean organizational commitment score was calculated by summing the individual scores and dividing by the maximum possible score. The mean overall percentage of organizational commitment was then calculated by multiplying the mean total score by 100. Based on the mean overall percentage, nurses' organizational commitment was classified as low (<60%), moderate (60–75%), and high (>75%) [32–34].

### 2.5. Instrument Validity and Reliability

A panel of five nursing administration academics from five nursing faculties at the universities of Benha, Ain Shams, Menoufia, Tanta, and Zagazig in Egypt evaluated the study instruments for their face and content validity. They evaluated the instrument items for conciseness, accuracy, completeness, and relevance. They approved the instrument tools after minor adjustments. Prior to actual data collection, a pilot study was conducted on 29 nurses, who were not included in the final statistical analysis of the study, to assess the feasibility, applicability, and clarity of the tools. According to this study, the estimated time to complete the questionnaire was 20–25 minutes.

The reliability of the study instrument was high, as revealed by Cronbach's alpha, with coefficients of 0.983, 0.986, and 0.996 for the knowledge, skills, and postcatastrophe management dimensions of the DPET, respectively, and 0.997, 0.991, and 0.995 for the affective, continuance, and normative commitment subscales of the OCS, respectively.

### 2.6. Fieldwork

Data were collected between July and the end of August 2023. The researchers provided nurses with a clear explanation of the study objectives and the process for completing the questionnaire. The head nurse of each unit scheduled the nurses' working hours based on their workload, and the researchers distributed the questionnaires and consent forms to the nurses at the scheduled times. The completed questionnaires were collected daily during the morning and afternoon shifts.

### 2.7. Ethical Considerations

This study received ethical approval from the Hail Research Ethics Committee (REC) (REC no. H-2023-316). The purpose of the study was clearly explained to eligible nurses before informed consent was obtained from those willing to voluntarily participate in the study. Nurses were informed that they had the right to withdraw from the study without consequences and that their data would only be used for research and analysis purposes. Nurses' privacy and the confidentiality of their data were strictly guaranteed.

### 2.8. Data Analysis

Data were coded and entered into Microsoft Excel spreadsheets for verification. They were then exported to IBM SPSS Statistics for Windows, version 25 (IBM Corp., Armonk, NY, USA) for subsequent analysis. Quantitative data were presented as mean and standard deviation (SD) for normally distributed data, or as median and interquartile range for nonnormally distributed data. Categorical data were presented as frequencies and percentages. The Mann–Whitney *U* test or Kruskal–Wallis H test was used to compare the median scores of nonnormally distributed data according to nurses' characteristics. In addition, Spearman's correlation was used to test the relationship between the mean total scores for knowledge, skills, and preparedness for postcatastrophe management and organizational commitment. Statistical significance was set at a *p* value of <0.05.

## 3. Results

### 3.1. Characteristics of the Respondent Nurses

The mean age of respondent nurses in this study was 32.1 ± 7.6 years, with most nurses aged 21–30 years (45.4%), followed by those aged 31–40 years (40.5%). The majority of nurses were females (81.1%), non-Saudi nationals (73.8%), and married (94.8%). Of the married and divorced nurses, 95.1% had children. Regarding qualifications, most nurses held a bachelor's degree in nursing or higher (72%), while the remainder had a diploma degree. The mean length of nurses' work experience was 9.7 ± 7.6 years (range: 1–28), with more than half having 1–9 years of experience. Most nurses were affiliated with the Maternity and Children's Hospital and King Khaled Hospital (35.0% and 29.7%, respectively) and reported no prior experience in catastrophe management (78.3%). On average, nurses cared for 6.0 ± 2.0 patients per shift and worked 8.7 ± 1.5 hours per day (Table 1).

### 3.2. Nurses' Preparedness for Catastrophe Management

The mean total scores for nurses' knowledge, skills, and preparedness for postcatastrophe management were 2.34 ± 1.26, 2.96 ± 1.18, and 2.88 ± 1.22, respectively, corresponding to a tendency toward disagreement (Table 2).

**Figure 1:**
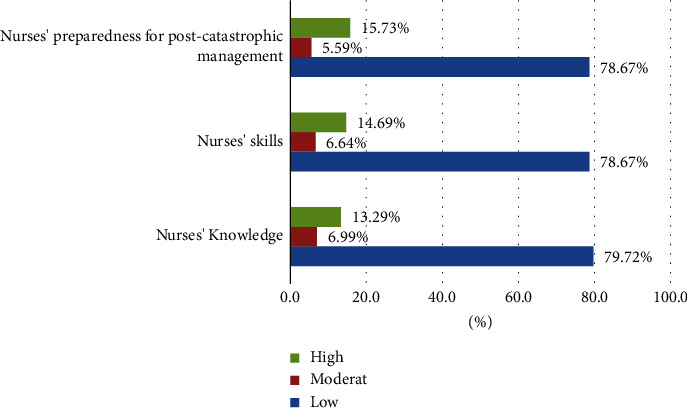
Preparedness levels for catastrophe management among nurses.

Regarding the level of preparedness, Figure 1 shows that most nurses had low levels of knowledge about catastrophe management (79.7%), compared with 7% and 13.3% for moderate and high levels of knowledge, respectively. Similarly, most nurses had low levels of catastrophe management skills (78.7%), compared with 6.6% and 14.7% for moderate and high levels of skills, respectively. In addition, low levels of preparedness for postcatastrophe management were observed in 78.7% of nurses, compared with 5.6% and 15.7% for moderate and high levels of postcatastrophe management, respectively.

### 3.3. Nurses' Organizational Commitment

The mean total scores for nurses' affective, continuance, and normative organizational commitments to their hospitals were 2.54 ± 1.25, 2.07 ± 1.36, and 2.39 ± 1.22, respectively, corresponding to a tendency toward disagreement. Furthermore, the level of organizational commitment was low, with a mean total score of 2.33 ± 1.22 (Table 3).

**Figure 2:**
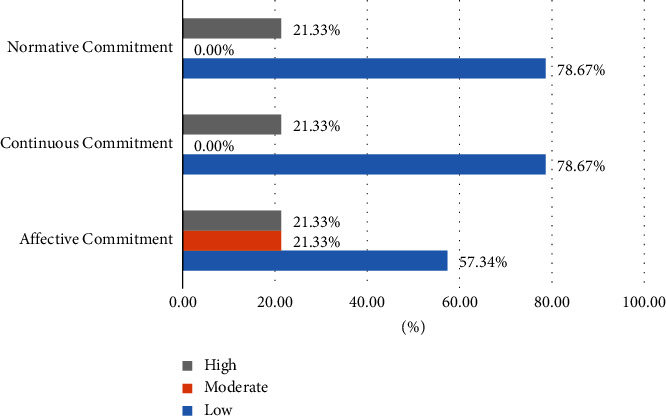
Levels of organizational commitment among nurses.

Regarding the levels of organizational commitment, Figure 2 shows that 57.3% of nurses had low levels of affective commitment to their hospitals, compared with 78.7% for continuance and normative commitments. In contrast, 21.3% of nurses demonstrated high levels of commitment to their hospitals on all three subscales of organizational commitment. However, moderate levels of affective commitment to hospitals were observed for 21.3% of the nurses, with no nurses demonstrating moderate levels of continuance or normative commitment.

### 3.4. Relationship between Nurses' Preparedness for Catastrophe Management and Their Organizational Commitment

Statistically significant positive, moderate correlations were found between nurses' knowledge and skills in managing catastrophes (*r* = 0.512; *p* < 0.01), knowledge and preparedness for postcatastrophe management (*r* = 0.492; *p* < 0.01), as well as nurses' skills and preparedness for postcatastrophe management (*r* = 0.533; *p* < 0.01). However, the nurses' level of organizational commitment was not significantly correlated with their knowledge, skills, or preparedness for postcatastrophe management (Table 4).

### 3.5. Differences in Nurses' Preparedness for Catastrophe Management and Organizational Commitment according to Their Characteristics

The median score for organizational commitment was significantly higher among single/divorced nurses (*P* = 0.001), nurses without children (*P* = 0.001), and those previously involved in real catastrophe management (*P* = 0.001) than among their counterparts. The median score of catastrophe management skills was significantly higher among nurses aged 21–30 years (*P* = 0.001) and nurses with 1–9 years of experience (*P* = 0.008) than among their counterparts, while the median score of postcatastrophe management was significantly higher among nurses aged 21–30 years and 31–40 years than those aged 40 years or older. However, no statistically significant differences were observed in the median knowledge scores according to nurses' characteristics (Table 5).

## 4. Discussion

By leveraging available resources and tools to prepare for catastrophes, catastrophe management provides a framework for managing the prevention and mitigation of negative consequences [37]. In this context, nurses must have the necessary knowledge and skills and be well-prepared to respond to and manage catastrophes effectively. Therefore, it is important to assess nurses' preparedness for catastrophe management and understand its relationship with their organizational commitment. To the best of our knowledge, this is the first study to assess the preparedness of nurses in public hospitals in Hail city in the northern region of Saudi Arabia for catastrophe management and its relationship with their organizational commitment.

The preparedness of nurses in the present study for catastrophe management was inadequate, as evidenced by their low levels of knowledge, skills, and preparedness for postcatastrophe management. This finding emphasizes the need for bolstering education and training in catastrophe management to improve their preparedness for responding to and managing potentially emerging catastrophes. As the likelihood of catastrophes in the future is on the rise, nurses should regularly undergo training in catastrophe management to ensure their skills remain up-to-date. The low preparedness of nurses could be due to the hospitals included in this study not prioritizing nursing training in risk management, considering that these hospitals are equipped with advanced technology aimed at protecting both patients and caregivers from potential risks. In accordance with the present study, it was found that most nursing students in Turkey lack the psychological and educational foundation required for responsible crisis response [14]. Similarly, Hasan et al. [37] found that nurses in Dhaka city, Bangladesh, had average levels of knowledge, skills, and catastrophe preparedness, suggesting that further training is needed to effectively manage catastrophes. In addition, nurses in Jordan and the Asia-Pacific region have been found to have low-to-moderate levels of preparedness for catastrophic events [31, 38].

This study also found nurses' low commitment to their hospitals, which may be attributed in part to their perception of being disconnected from these hospitals due to their inability to engage in decision-making and fewer opportunities to interact directly with supervisors. In addition, hospitals may lack clear communication channels in this regard. Many nurses may also have little experience and lack emotional attachment to their hospitals. This finding contradicts the finding of Elzohairy et al. [39], who found that professional nurses in Damanhur, Egypt, had moderate organizational commitment. Moreover, Callado et al. reported that primary healthcare nurses were highly committed to their organizations [40].

The significant positive moderate correlation between nurses' knowledge and their skills in managing catastrophes in the present study suggests a logical direct relationship between these two factors. In this regard, more knowledgeable nurses tend to be more skilled at dealing with catastrophic situations, reflecting the importance of nursing education in catastrophe management to improve nurses' ability to respond to and manage catastrophes effectively. This finding is consistent with the findings of Hasan et al. [37], who revealed such a relationship among nurses in Dhaka city. Furthermore, the significant positive moderate correlation between nurses' knowledge and preparedness for catastrophe management in the present study could be attributed to the willingness of knowledgeable nurses to learn, practice, and acquire the skills required to deal with catastrophic situations. This finding also agrees with that reported among nurses in Dhaka city [37]. On the other hand, the nurses' level of skills in the present was significantly and positively correlated with their preparedness for postcatastrophe management, suggesting that better skills are linked to higher preparedness. This finding agrees with that reported among nurses in Dhaka city [37].

Despite not finding a significant correlation between nurses' preparedness for catastrophe management and their organizational commitment, it is possible that this commitment still indirectly affects their preparedness. Various factors, including emotional attachment to their hospitals, involvement in decision-making, direct interaction with supervisors, and clear communication channels within the hospital, influence nurses' organizational commitment. Nurses who are committed to their organizations may be more likely to pursue professional development that can improve their catastrophe management knowledge and skills. In contrast to the present finding, a positive correlation was found between Indonesian nurses' organizational commitment and their preparedness for dealing with patients during natural catastrophes [22].

The present study found that organizational commitment increased significantly among single or divorced nurses, those who had no children, and nurses with prior involvement in real catastrophe management. It is plausible that the fewer family-related obligations or distractions of these nurses may allow them to dedicate more time to their organizational goals, enhancing their organizational commitment. On the other hand, the struggle to balance work and family obligations can often dilute nurses' commitment to their organizations. Personal circumstances, such as marriage and childcare responsibilities and preoccupation with family matters can negatively influence nurses' commitment to their organizations. In contrast, a previous study in Saudi Arabia found that married nurses had significantly higher levels of organizational commitment [41].

Nurses' level of skills in the present study increased significantly by their age and experience. Similarly, nurses with less than three years of experience in Saudi Arabia were found to need more catastrophe management training [7]. On the other hand, the increase in nurses' organizational commitment because of their experience in catastrophe management could be attributed to the fact that the acquired nurses' experience enhances their loyalty to the organization by deepening their understanding of institutional policies, regulations, and procedures. Consequently, greater experience is associated with greater organizational commitment. In contrast to the present study, Alammar et al. [42] found no significant difference in Omani nurses' organizational commitment according to their years of experience. However, the present study found a decrease in nurses' preparedness for catastrophe management with their age increase, which is contrary to the common belief that learning and cognitive skills for education increase with age. This finding is consistent with previous studies among nurses in Japan and Iran [43, 44].

This study provides valuable information about the preparedness of nursing staff in Hail city for catastrophe management. However, there are several limitations that may affect the interpretation of the results. The cross-sectional correlational design of this study makes it difficult to establish a causal relationship between nurses' preparedness for catastrophe management and their organizational commitment, as well as other potential influencing factors. In addition, restricting the study to public hospitals and relying on convenience sampling also contribute to the limited generalizability of the results to the broader nursing population in the city. It is worth mentioning that nurses in the private sector may face different situations, working conditions, and organizational structures that may lead to different scenarios regarding the topic under study. Therefore, large-scale studies with more representative samples, along with longitudinal designs, are recommended to assess the relationship between nurses' preparedness for catastrophe management and their organizational commitment.

### 4.1. Implications for Practice, Research, and Education

Greater commitment of nurses to their hospitals increases the likelihood of adequate preparedness to deal with catastrophic events effectively. Therefore, hospitals should prioritize improving the organizational commitment of their nursing staff by fostering a supportive work environment. In addition, it is crucial to implement strategies that strengthen nurses' attachment to their hospitals, such as offering opportunities for professional development and recognizing the positive contributions made by nurses. This study emphasizes the importance of integrating catastrophe management into nursing curricula and programs to improve nurses' knowledge and skills in preparing for and responding to catastrophes and postcatastrophic situations. Hospital administrators should also provide nurses with continuous education and regular training programs to keep them up-to-date and improve their capability to handle catastrophic events. The findings of this study warrant further studies to identify and predict potential factors affecting nurses' preparedness for catastrophe management, as well as the development of effective strategies that ensure a more efficient response and management of catastrophic events.

## 5. Conclusion

Nurses in Hail city are not adequately prepared to respond to and manage catastrophes and postcatastrophic situations, and they have low organizational commitments to their hospitals. Therefore, nursing education should integrate catastrophe management into the curricula, and hospital administrators should prioritize a supportive work environment that strengthens organizational commitment and provides ongoing education and regular training to improve nurses' preparedness for catastrophe management.

## Figures and Tables

**Table 1:** Characteristics of respondent nurses included in the study (*N* = 286).

Characteristics	*n* (%)
Age (years)	
21–30	130 (45.5)
31–40	116 (40.5)
≥41	40 (14.0)
Mean ± SD	32.1 ± 7.6
Gender	
Male	54 (18.9)
Female	232 (81.1)
Nationality	
Saudi	75 (26.2)
Non-Saudi	211 (73.8)
Marital status	
Single/divorced	15 (5.2)
Married	271 (94.8)
Having children	
No	14 (4.9)
Yes	272 (95.1)
Qualification	
Diploma	80 (28.0)
Bachelor's or higher	206 (72.0)
Length of professional experience (years)	
1–9	150 (52.4)
10–19	105 (36.7)
20–28	31 (10.8)
Mean ± SD	9.7 ± 7.2
Hospital affiliation	
Hail General Hospital	46 (16.1)
King Salman Hospital	55 (19.2)
King Khaled Hospital	85 (29.7)
Maternity and Children's Hospital	100 (35.0)
Involvement in real catastrophe management	
No	224 (78.3)
Yes	62 (21.7)
Number of patients per shift	
Mean ± SD	6.0 ± 2.0
Daily work hours	
Mean ± SD	8.7 ± 1.5

SD, standard deviation.

**Table 2:** Preparedness for catastrophe management among nurses.

Preparedness dimension	Mean score ± SD^∗^	Direction
Knowledge	2.34 ± 1.26	Disagree
Skills	2.96 ± 1.18	Disagree
Preparedness for postcatastrophe management	2.88 ± 1.22	Disagree

^*∗*^The total number of respondent nurses was 286. SD, standard deviation.

**Table 3:** Organizational commitment of nurses.

Commitment subscale	Mean score ± SD^∗^	Direction
Affective commitment	2.54 ± 1.25	Disagree
Continuance commitment	2.07 ± 1.36	Disagree
Normative commitment	2.39 ± 1.22	Disagree
Level of commitment	2.33 ± 0.82	Low

^*∗*^The total number of respondent nurses was 286. SD, standard deviation.

**Table 4:** Correlation between nurses' preparedness for catastrophe management dimensions and organizational commitment.

Items	Knowledge	Skills	Postcatastrophe management	Organizational commitment
Knowledge	1			
Skills	0.512^*∗∗*^	1		
Postcatastrophe management	0.492^*∗∗*^	0.533^*∗∗*^	1	
Organizational commitment	0.011	−0.010	0.031	1

^*∗∗*^Statistically significant (*p* < 0.01).

**Table 5:** Differences in preparedness for catastrophe management dimensions and organizational commitment among nurses according to their characteristics.

Variables	Knowledge			Skills		Preparedness for postcatastrophe management		Organizational commitment	
	*N*	Median (IQR)	*p* value	Median (IQR)	*p* value	Median (IQR)	*p* value	Median (IQR)	*p* value
Gender									
Male	54	2.08 (1.52)	0.960	2.18 (0.84)	0.64	2.57 (0.95)	0.59	2.00 (2.33)	0.20
Female	232	2.08 (2.00)		2.82 (0.98)		2.57 (1.00)		1.92 (0.44)	
Marital status									
Single/divorced	15	2.08 (1.69)	0.68	3.00 (0.82)	0.69	2.09 (1.14)	0.35	4.00 (2.89)	**0.001**
Married	271	2.08 (2.00)		2.82 (0.91)		2.57 (1.00)		1.94 (0.44)	
Nationality									
Saudi	75	2.08 (1.69)	0.83	2.18 (1.00)	0.87	2.57 (1.00)	0.73	1.78 (0.44)	0.33
Non-Saudi	211	2.08 (2.00)		2.82 (0.91)		2.52 (1.00)		2.0 (0.67)	
Having a child									
No	14	2.08 (1.69)	0.96	2.59 (0.86)	0.96	2.17 (1.00)	0.23	4.00 (2.97)	**0.001**
Yes	272	2.08 (2.00)		2.82 (0.91)		2.57 (1.00)		1.94 (0.44)	
Qualification									
Diploma	80	2.08 (2.46)	0.08	3.00 (2.61)	0.28	2.57 (2.40)	0.17	2.00 (0.67)	0.53
Bachelor's or higher	206	2.08 (1.52)		2.18 (0.91)		2.55 (0.96)		1.89 (0.44)	
Involvement in real catastrophe management									
No	224	2.08 (2.00)	0.92	2.50 (1.00)	0.70	2.57 (1.00)	0.32	1.72 (0.50)	**0.001**
Yes	62	2.08 (1.69)		2.81 (0.82)		2.31 (1.00)		4.61 (0.94)	
Age (years)									
21–30	130	2.08 (2.50)	0.13	3.00 (2.02)	**0.001**	2.75 (2.15)	**0.016**	1.83 (0.44)	0.497
31–40	116	2.08 (1.08)		2.18 (1.00)		2.75 (1.00)		1.97 (0.63)	
≥41	40	2.08 (1.31)		2.18 (1.00)		2.00 (0.54)		2.00 (1.92)	
Length of work experience (years)									
1–9	150	2.08 (2.00)	0.34	3.00 (0.82)	**0.008**	2.52 (0.96)	0.35	1.89 (0.44)	0.69
10–19	105	2.08 (1.31)		2.18 (1.00)		2.57 (1.00)		1.94 (1.50)	
20–28	31	2.31 (2.38)		2.82 (2.09)		2.00 (2.10)		2.00 (0.67)	
Hospital affiliation									
HGH	46	2.08 (3.46)	0.08	3.00 (2.36)	**0.001**	2.57 (2.63)	**0.007**	1.67 (0.57)	**0.019**
KSH	55	2.08 (1.08)		2.18 (1.00)		2.52 (0.76)		2.00 (0.61)	
KKH	85	2.08 (1.08)		3.00 (0.82)		2.09 (0.95)		1.72 (2.39)	
MCH	100	2.08 (2.37)		3.00 (2.07)		2.67 (2.12)		2.00 (0.44)	

IQR, interquartile range; HGH, Hail General Hospital; KSH, King Salman Hospital; KKH, King Khaled Hospital; MCH, Maternity and Children's Hospital. The bold values indicate *p* < 0.05.

## Data Availability

The data used to support the findings of this study are available from the corresponding author upon request.
